# Feeling Offended: A Blow to Our Image and Our Social Relationships

**DOI:** 10.3389/fpsyg.2017.02221

**Published:** 2018-01-17

**Authors:** Isabella Poggi, Francesca D’Errico

**Affiliations:** Dipartimento di Filosofia Comunicazione e Spettacolo, Universitá degli Studi Roma Tre, Rome, Italy

**Keywords:** feeling offended, socio-cognitive model of emotions, social emotions, gender differences, self-esteem

## Abstract

The paper presents a survey study that investigates the self-conscious emotion of feeling offended and provides an account of it in terms of a socio-cognitive model of emotions. Based on the qualitative and quantitative analysis of the participants’ answers, the study provides a definition of offense and of the feeling of offense in terms of its “mental ingredients,” the beliefs and goals represented in a person who feels this emotion, and finds out what are its necessary and aggravating conditions, what are the explicit and implicit causes of offense (the other’s actions, omissions, inferred mental states), what negative evaluations are offensive and why. It also shows that the feeling of offense is not only triggered about honor or public image, but it is mainly felt in personal affective relationships. The paper finally highlights that high self-esteem may protect a person against the feeling of offense and the constellation of negative emotions triggered by it.

## Introduction

In social psychology research, the feeling of offense has been viewed so far as typically triggered by a blow to a person’s honor, hence to his/her public “face”; yet this painful emotion, beside nicking the reputation and self-concept of the offended person, is often felt also in interpersonal relationships, that it finally may seriously disrupt.

Feeling offended belongs to the so-called “self-conscious emotions” (Lewis, 2008), like shame, guilt, and pride, and like shame and humiliation it is caused by a blow to the person’s image and self-image. The self-conscious emotions, traditionally opposed to “basic” ones (Darwin, 1872; Ekman, 1982), are far less studied; but are they really less “basic” than them?

This paper argues that research on self-conscious emotions should be enhanced; and tries to contribute to this endeavor by presenting a study on the feeling of offense.

Our aim is to provide a theoretical definition of this emotion and its necessary conditions, and an empirical analysis of its triggering events, the contexts and situations in which it is felt, and other feelings connected to its occurrence. In doing so, we stress that the feeling of offense does not only dwell in the field of honor and public image, as implied by previous research, but it mainly affects our close relationships, any time an action or omission of others challenges our sense of personal value and disrupts our reciprocal relations. Our primary goals are to obtain a real-life account of the feeling of offense, and to understand its connection with the person’s sense of self and her social and affective relationships.

The Section “Related Studies and Research Questions” overviews current research on offense and the sense of honor. The Section “Feeling Offended: Research Goals” lists some still open-research questions, while the Section “A Socio-cognitive Model of Image, Self-Image, and Their Monitoring Emotions” presents a socio-cognitive model of mind and social interaction adopted to frame the research, introducing the notions of emotion, evaluation, image and self-image, shame, and pride, to pave the way for a conceptual definition of the feeling of offense. This top-down characterization is then verified in the empirical study presented in the Section “Feeling Offended: a Research Study,” that investigates contexts, reasons, functions of this emotion, thus contributing to single out its “mental ingredients,” i.e., the beliefs and goals represented in the mind of a person who is feeling offended, and to establish its relations with personal factors like gender and self-esteem. Conclusions follow in the Section “Conclusion.”

## Related Studies and Research Questions

Feeling offended is a complex emotional state involving *personal factors* (gender, self-esteem) that can modulate it on the basis of different expectations or causal attributions (internal vs. external); but it also involves *relational factors* that affect the interpretation of the offense, since the “offenders” can be relatives, friends, acquaintances, co-workers, each implying different emotional costs. As to the personal factors, self-esteem plays a crucial role in the feeling of offense, since it can affect self-relevant emotions like shame and pride (Brown and Marshall, 2001): people with low self-esteem tend to feel shame more than others. Gender mediates the feeling of offense mostly in familiar contexts (Mosquera et al., 2002).

The multidimensional factors that characterize this feeling have been investigated in various psychological fields, from the dynamic approach to social psychology.

According to Zander (1976), the feeling of offense is a profound emotional state which goes through three phases: (1) identification of the cause, interpreted as an insult to an ideal value; (2) feeling of offense, with its relative intensity related to the “expectations of recognition,” and (3) reaction to the feeling of offense, also taking into account “socio-historical variables.”

In the socio-cognitive field, Mosquera et al. (2002) tested how feeling offended can be referred to honor cultures (specifically, they consider Spain vs. Netherlands), where typically offenses take place in public or are referred to masculinity or female sexual morality. Cohen et al. (1996) emphasize that, the higher the honor concern and the significance of the honorable person, the strongest the emotional response to insults. In cultures with a stronger code of honor, like Spain, there are more embarrassed reactions, mostly in relation to threats to family honor, so important for individual self-esteem that when someone is offended one’s own self-esteem can be damaged too. Actually, some circularity affects these studies, since self-esteem is defined as what is affected by offenses. Furthermore, the study reports gender differences in emotional reactions of both shame and anger in case the insults undermine the sexual dimension, especially with Spanish women, for whom the sexual code of honor (sexual shame) is stronger than for Duch women.

A problem in these studies is that they mainly investigate the emotional responses triggered by explicit offenses, with particular attention to verbal insults, while neglecting offenses that are less direct, less explicit, and associated to personal rather than public factors.

Interpersonal and intergroup elements are central to the feeling of offense in studies on forgiveness (McCullough, 2000; Paleari et al., 2005): those who feel offended may feel inferior in terms of perceived control (Baumeister et al., 1994) and experience feelings of victimization or anger (McCullough et al., 1998), which results in a need to restore their sense of power, by also increasing power-seeking behavior (Foster and Rusbult, 1999).

These needs are welcome within a possible socio-emotional reconciliation perspective in which the offender attempts to undertake a “cycle of apology-forgiveness” (Nadler, 2002): the responsible person puts her personal image in the hands of the other person, at the risk of not being forgiven. This presupposes a willingness to forgive as a result of a long-term reconciliation path, for example where the transgressor admits one’s responsibilities.

Studies on forgiveness and reconciliation provide a complex framework where personality, ruminating tendencies, emotional stability, empathy toward the transgressor can lead the offended person to forgive, if offenses are explicit (e.g., betrayal, physical, and emotional humiliation). Yet, to our knowledge, no studies have analyzed how feeling offended can be caused by implicit acts (for instance, not insults), nor cases in which the social relationship or personal investment (then the resulting expectations) can exacerbate the emotional reactions following the offense.

## Feeling Offended: Research Goals

The quoted works on the feeling of offense have always attributed the causes of this mental state to an explicit action, strictly connected to honor issues, as in the case of insults (Mosquera et al., 2002). Yet sometimes we feel offended also for more subtle and implicit causes: a simple lack of attention on the part of the other, or his refusal of our offer of help; or even an altruistic behavior by him, which makes us feel helpless and hence humiliates us.

Therefore, the first aim of our work is to provide a more comprehensive definition of feeling offended considering all possible types of offenses, both public and personal, both explicit and implicit: actions, communicative acts, and inferred mental states.

Second, specific new questions and ones stemming from the above literature will be addressed: what are the specific effects of different causal attributions or expectations on this emotional state, and do they vary according to different social relationships, namely with relatives, friends, colleagues, or strangers? What are the emotions typically triggered by the “feeling of offense” in different types of social/affective relationships? What is the role of personal factors such as gender and self-esteem in mediating the emotional processing preceding and following the feeling of offense? And what is the role of relational factors in affecting the intensity of this emotion and its possible consequences? A final objective or our study is to deepen the role of self-esteem, as a personal variable working as a possible “buffer” (Andt and Goldenberg, 2002) to negative consequences of feeling offended. Discovering the link between self-esteem and feeling offended will also allow us to better explore the role played by gender differences in this emotional state, given that, for personal (as shown by Baumeister et al., 2003) but also cultural and historical reasons (Faniko et al., 2015; Bleidorn et al., 2016), women report lower levels of self-esteem than men.

## A Socio-Cognitive Model of Image, Self-Image, and Their Monitoring Emotions

In this section we overview the socio-cognitive model of mind, social interaction, communication, and emotions in terms of goals and beliefs that we will adopt to investigate the research questions above.

According to Parisi and Castelfranchi (1975), the life of any natural or artificial, individual, or collective system consists of pursuing *goals*: regulatory states that, when not realized in the world, trigger plans, hierarchical structures where each action aims at a goal and possibly to superordinate supergoals. In this framework, *power* is first defined as a notion concerning a single system, “*power of*,” the likeliness to achieve one’s goals, which depends on favorable world conditions (e.g., presence of material resources), and the system’s action capacities and knowledge. If system A lacks capacities or resources to achieve a goal g, while another Agent B is endowed with them, A *depends on* B and can achieve g only if B *adopts* A’s goal g, i.e., if B puts one’s actions and resources to the service of A’s goal. The device of *adoption* multiplies people’s power to achieve goals, thanks to resource exchange; but, in order to decide whose goals to adopt, people need to evaluate others as to their dependence on them and to their capacity and willingness to reciprocate: people form an *image* – a set of evaluative and non-evaluative beliefs – about others. An *evaluation* is defined in terms of “*power of*”: a belief about how much some object, event, person have or provide one with the “*power of*” necessary to some goal. Evaluations about world conditions, adequacy of actions, respective importance of goals are necessary in both deciding which goals to pursue and making plans to achieve them. People judge others and themselves in terms of various criteria: *aesthetic* criteria (beauty), but also *competence* (cognitive skills, reasoning, memory, planning capacity), *benevolence* (altruism, empathy, care for others, honesty, sincerity), *dominance* (strength, assertiveness, persuasiveness, leader skills) (D’Errico and Poggi, 2012).

We generally strive to present a positive image to gain others’ esteem and have positive relations with them (to have them adopt our goals): we have a “*goal of image*” – the goal of having others make up an image of us – and a “*goal of esteem*” (positive image). But different people may want to be evaluated positively against some specific goals – I strive to appear a charming woman, you prefer to look a knowledgeable scholar. Further, we have a *self-image*, a set of evaluative and non-evaluative beliefs about ourselves, necessary to decide which goals to pursue, leaving aside ones out of our reach; and since having positive self-evaluations (a high *self-esteem*) gives us confidence in pursuing our goals, we also have a *goal of positive self-image*. A person’s image and self-image are tightly connected since they determine each other (Mead, 1934), but a person’s adaptation mainly depends on her self-confidence, which is especially preserved when her self-image is not too dependent on the image other people have of her. To achieve an independent judgment, a person makes up her own set of values, the criteria of evaluation with respect to which she will evaluate herself in order to her positive self-image, and sticks to them even if they are not the same against which others evaluate her.

Preserving a good image and self-image are among the most important goals of a person, being a means to gain adoption. Yet, sometimes people cast discredit over us, that is, they try to spoil our image by finding out our (real or supposed) flaws, and spreading negative evaluations about us. Discrediting a person means to spoil her image before some audience, inducing others to believe s/he is not so good, beautiful, smart, powerful as s/he tries to appear (D’Errico and Poggi, 2012; Poggi et al., 2015), by communicative acts of criticism or accusation, that highlight a wrong action by the target, or by insults, which evidence a severe flaw of it (e.g., *stupid*) or claim its belonging to a degrading category (*pig*).

In this framework, emotions are seen as multifaceted subjective states, encompassing internal feelings, cognitive, physiological, expressive, motivational aspects, that are triggered any time an important adaptive goal of ours is, or is likely to be, achieved or thwarted (Castelfranchi, 2000; Miceli and Castelfranchi, 2014). They are functionally linked to human adaptation (Frijda, 1986; Scherer, 2009), monitoring the achievement or thwarting of goals like survival and wellbeing, acquisition of knowledge, acquisition, and maintenance of resources, but also the goals of equity, attachment and affiliation, image, and self-image (Poggi, 2008). We feel positive emotions for the achievement and negative ones for the thwarting of these goals; hence, emotions can be clustered according to the type of goal they monitor. Fear, felt when our physical safety is at risk, monitors the goal of survival, boredom, experienced in absence of new stimuli, monitors the goal of continuous acquisition of novel knowledge.

An important subset of emotions is “social emotions” (D’Errico and Poggi, 2016): those felt toward another person – like envy or compassion – or importantly connected to our relationships with others. Among these are the self-conscious emotions, that monitor the goals of image and self-image, like pride and shame: in fact, we feel shame when we think that how we are or what we do may cause others or ourselves to have a negative image of us (Castelfranchi and Poggi, 1990), we feel pride for a positive image or self-image (Poggi and D’Errico, 2011).

The socio-cognitive framework analyses the cognitive aspects of emotions in terms of their “mental ingredients,” that is, the beliefs and goals represented in our mind when we feel a given emotion: e.g., pride entails, among others, the ingredients ACTION, PROPERTY, CAUSE, POSITIVE EVALUATION, SELF-IMAGE.

A relevant issue in pride and shame, both linked to image and self-image, is that generally the sharing of values between Self and Other is a necessary conditions for feeling the emotion, while factual knowledge is not.

In Hitchcock’s film “I confess,” father Logan (Montgomery Clift) is suspected by a murderer to have revealed his murder that he had confessed. Logan does know he did not reveal it, but the murderer does not. Notwithstanding this, as beautifully expressed by Clift’s intense interpretation, Logan feels highly painful shame because, though not sharing factual belief, he shares the value that a priest must not reveal the content of a confession.

In the remainder of the paper, we present a survey study investigating the feeling of offense with the aim of finding out the “mental ingredients” of this emotion, stating its relationship with the goals of image and self-image, and thus integrating a deeper knowledge of this affective state into the model above.

## Feeling Offended: A Research Study

The goal of our work, that we pursue through both a top-down and a bottom-up approach, is twofold: on the theoretical side we want to provide a definition of the feeling of offense in terms of the socio-cognitive model above, singling out its mental ingredients and stating its connection with the goals of image and self-image; on an empirical side, by means of a survey study we want to answer the research questions above, concerning the causes and effects of this feeling, while testing the hypothesis that the tendency to feeling offended is increased by lower self-esteem.

We first propose a definition of the feeling of offense in terms of our socio-cognitive model; then, we present an empirical study aimed at testing and deepening our definition.

### Research Questions and Working Hypotheses

An offense is a wound, an injury to the soul^1^, an attack to something even more important than the integrity of our body: our image. We feel offended every time we think that someone conceives – and possibly communicates to ourselves or others – an evaluation of us that is worse/lower than one we think we deserve. Yet, this wound is particularly serious since it does not only sully the image that the offender or others have of us, but nicks an even more precious good of ours: our self-image. The offense insinuates the doubt that what others attribute to us is a real flaw of ours.

Since the goals of image and self-image are so adaptively important for our life, the moral inury of an offense triggers a very painful emotion: we feel offended.

In our hypothesis, the feeling of offense is a negative emotion felt when an action or omission of someone, with whom we have a relevant affective relationship, causes a blow to our image and, possibly, self-image. Our survey study aims at singling out the “cognitive ingredients” of this emotion. Further research questions concern the causes, conditions, social and cognitive mediators of the feeling of offense, the connected emotions, and the relationships between the offender and the offended person; finally, our hypothesis is that the tendency to feeling offended is increased by lower self-esteem.

### Participants and Methods

To address these issues, we designed a semi-structured online survey on “feeling offended” that investigated the features and the effects of this feeling through the recalling of autobiographical episodes (Goodwin and Williams, 1982). The survey was submitted to a sample of 129 participants, mainly Italians, balanced and composed by 61% women (n. 79, vs. 50 males), age 31.2 years (*SD* = 14.1), the majority with a high school bachelor (54%) or a University degree (26%).

The survey included 14 closed and 11 open questions, asking how frequently the participants felt offended, for what reasons, who offended them, and in what life domain (work, family, friends, etc.). To go more into the emotional experience of feeling offended, participants were asked to report one case in which they felt so, the specific reasons why they did, if they believed the other intended to offend, their relationship with the other before and after the offense, and what other emotions they connected to the feeling of offense. We also asked if there was some case in which another wanted to offend them but they did not feel offended, and if so, why they did not; and conversely, if in some cases another person had felt offended by them, but should not have felt so, and why. Finally, we asked to provide a definition of “feeling offended.”

After this questionnaire, participants filled in Rosenberg’s test of self-esteem (Rosenberg, 1965). A content analysis was conducted on the open questions, and a data analysis of personal (gender and self-esteem) and relational factors (type of relations) of the measured variables.

### Results: Cognitive Ingredients, Causes, and Conditions of Feeling Offended

The open questions subject to qualitative analysis concerned the definition of feeling offended provided by participants, the typical situations in which one feels offended, and the specific events that are felt as offending. From the analysis, some differences result sometimes between the mental ingredients explicitly mentioned by our participants in their definition of this emotion and those explicitely or implicitly present or implied in their reported examples. Let us start with the participants’ definitions.

#### “Feeling Offended” in Participants’ Definitions

Looking for the ingredients that, all in all, are contained (mentioned or alluded to) in participants’ definitions, in their words the feeling of offense appears as a negative emotion felt by A, often close to or embedding humiliation, anger, bitterness, sadness, rancor, the feeling of being misunderstood, impotence, and annoyance. It is caused by either a non-communicative or a communicative act by B that results into an aggression to A’s image, since it explicitly points at or implicitly entails a negative property of A: a property worth a negative evaluation of A by B with respect to an evaluation criterion relevant for the image which A wants to project, and shared with B. Being attributed this negative property is seen as a true wound to A’s image [the root *feri-* of verb *ferire*, adjective *ferito*, and noun *ferita* (wound, wounded) occurs as much as 41 times]. This wound somehow implies a lack of respect for A (lack of care for his/her image), and the aggression is considered unjust by A, A thinks s/he does not really deserve to be attributed such a property; a misunderstanding or unwarranted assumption by B holds such that A, though sharing the evaluation criterion with B, does not share the same factual knowledge: A and B share the value in terms of which facts can be judged, but not the really occurred facts. The problem with A is that B is relevant for A, since keeping a positive social relationship with B is important for A.

The whole fact results in subsequent negative social emotions of A toward B, such as disappointment and feeling betrayed by B, finally ending with a break in the social relationship of A with B, but also with a loss of self-esteem for A.

Following are the emotions, close or embedded within the feeling of offense, evoked in participants’ definition of offense, or in the description of its feelings:

*Humiliation* is explicitly mentioned in both definitions and descriptions of feeling offended, sometimes coupled with it, and humiliating someone is seen as the goal of offending him.

*Anger* is mentioned rarely in participants’ definitions but 30 times in their descriptions, with connected emotions like disappointment, but also with antagonistic ones like sadness and shame. Further mentioned emotions are *bitterness* and *rancor*, with the latter often seen as a final result of feeling offended, and the reason why the offense results in a break of the social relationship between A and B.

The ingredients of feeling offended are quite clearly phrased in participants’ definitions. Some focus on its being a negative feeling due to some communicative or non-communicative action by a person with whom one wants to maintain a positive relationship:

5: *rimanerci male rispetto a un qualcosa detto o fatto da una persona alla quale tieni:*
(to get it wrong due to something said or done by a person you care)

NEGATIVE EMOTION (*rimanerci male*)
CAUSED BY A COMMUNICATIVE ACTION (*rispetto a un qualcosa detto*)
OR A NON-COMMUNICATIVE ACTION (*o fatto*)
BY A PERSON RELEVANT FOR A (*da una persona alla quale tieni*)

Some specify that the NEGATIVE EMOTION is (caused by) a wound to their own identity, dignity, or pride, caused by an AGGRESSION on the part of someone you esteemed, conveying a NEGATIVE EVALUATION of A that is generally considered UNJUST by A: this implies that A, though sharing the same criterion of evaluation, does not share the same factual knowledge as B, and claims being accused with no guilt: NO FACT SHARING.

Yet, in few cases – typically concerning physical appearance – A feels offended just because s/he does share factual knowledge.

The negative evaluation is felt as a WOUND and a BETRAYAL:

32: *offendere è come un pugnale tra le scapole. Più l’offesa è grave più il coltello va affondo e ti ferisce.*
(offending is like a dagger in your shoulder blades. The more severe the offense, the more the dagger goes deep and injures you).

resulting in LACK OF RESPECT and HUMILIATION of A and causing ANGER and RANCOR against B. Some participants stress the IMPORTANCE OF THE RELATIONSHIP WITH B, and A’s PREVIOUSLY POSITIVE RELATIONSHIP WITH B (even esteem), which causes A’s DISAPPOINTMENT about B’s action. For others, the very definition of offense is in terms of its internal and social consequences: a LOWERED SELF-ESTEEM of A, and a BREAK OF THE RELATIONSHIP WITH B.

These ingredients of offense are explicitly mentioned in participants’ definitions, but in their narratives the offense is often caused by a NON-ACT: AN OMISSION. Let us see what actions or non-actions may be offensive.

#### What Is the Cause of the Offense?

**FIGURE 1:**
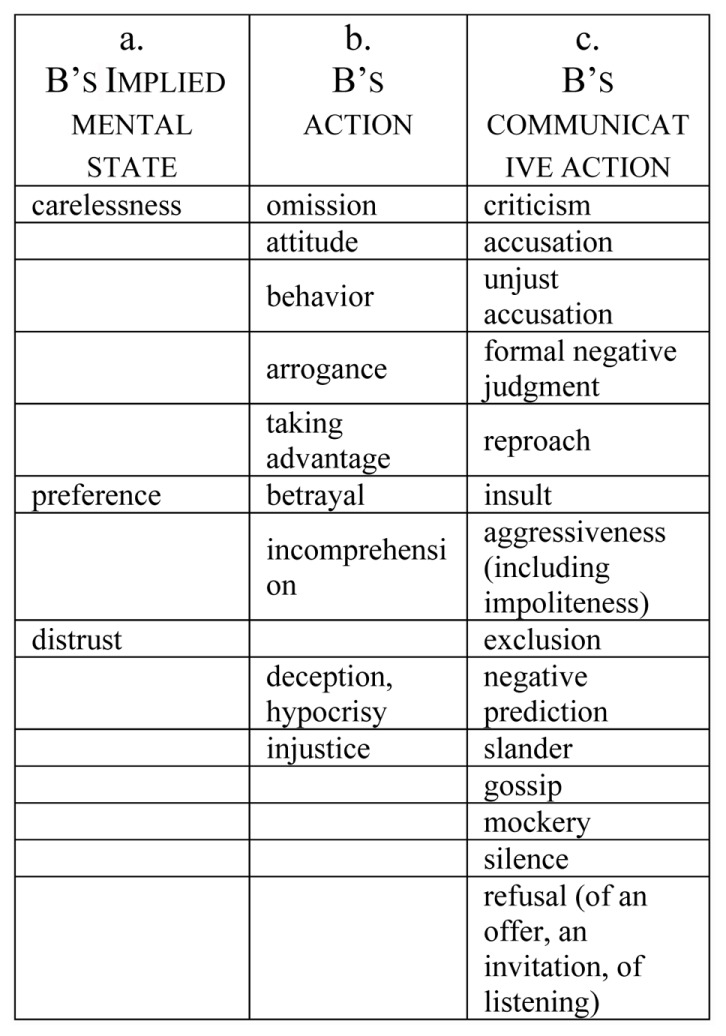
Causes of offense.

**Figure 1** represents the causes of offense resulting from participants’ definitions and storytellings.

(a) Feeling offended by an IMPLIED MENTAL STATE

What offends A are most typically communicative actions (col. c); but B’s non-communicative actions can be offensive too (col. b); finally, sometimes A is offended not by what B does or does not do, but by an implicit mental state of B that can be indirectly inferred from B’s communicative or non-communicative behavior (col. a). Let us start with this indirect cause of offense.

16: *Quando ho dato dei consigli a dei familiari ma non mi hanno ascoltato e si [sono] fidati di altri, i quali hanno fornito le mie stesse opinioni.*
(when I gave advice to relatives but they did not listen to me and trusted others, who provided the same opinions).

Here what is offensive for A is a substantive *distrust* of B for A that is made explicit by B’s not following A’s advice.

Another very offensive mental state, generally implied by an omission, is the other’s *carelessness*:

52: *Una mia cugina non ha mantenuto la sua promessa di venirmi a trovare, e non mi ha più cercato.*
(A cousin of mine did not keep her promise to come visit me, nor did look for me anymore).

That the other disregards her own promise means that you are not important for her, she does not care you and your feelings: something highly upsetting. Further, if your low importance for the other is a bad hit to your self-esteem, even more so is the comparison between how important you are for the other as opposed to other people. Thus when the other *prefers* someone else over you, you feel betrayed: and betrayal is not only offensive *per se* but mostly because A finally loses in the comparison between him/her and the rival, who is preferred by B. Like in this example:

112: *mia sorella si è sposata e non mi ha voluto come testimone dopo che me l’aveva già chiesto.*
(my sister got married and did not want me as her wedding-witness, after she had asked me to).

In these cases B’s preference uncovers A’s relative unimportance; but the extreme case is not being acknowledged at all as a person, for example, not being greeted when meeting others.

(b) Offended by NON-COMMUNICATIVE ACTIONS

Sometimes, what is offensive is not a particular communicative behavior, but a general attitude of B toward A:

35: *Quando frequentavo l’università, una mia insegna[n]te, nonché relatrice, spesso mi faceva sentire un’ignorante.*
(As I attended the university, a teacher of mine, and tutor, often made me feel an ignorant person).

Another offensive behavior is when B “takes advantage” of A: this makes A feel “used” like an object, not credited the dignity of a human person with her personal goals and desires.

Finally, injustice is offensive:

27: *A lavoro quando non mi è stato riconosciuto il merito di un compito svolto.*
(At work when I was not acknowledged the merits of a task performed).

Being subject to injustice is offensive also for an underlying thought: how unworthy am I so as to be treated this way?

(c) Offensive COMMUNICATIVE ACTIONS

A person can be offended by a criticism, a slander, an unjust accusation; by gossip, insults, mockery, but also by a reproach, a formal negative judgment (like a bad score), a negative prediction:

107: *quando a venti anni mi dicevano che non avrei fatto molto nella vita.*
(when at 20 people told me I would not do so much in my life).

The bulk of offensive action is exclusion

29: *quando un professore mi ha cacciato da un esame orale.*
(when a teacher sent me away of an oral examination).

which can take the form of a refusal to listen, or of the other’s silence.

#### What Negative Evaluations Are Offensive?

**FIGURE 2:**
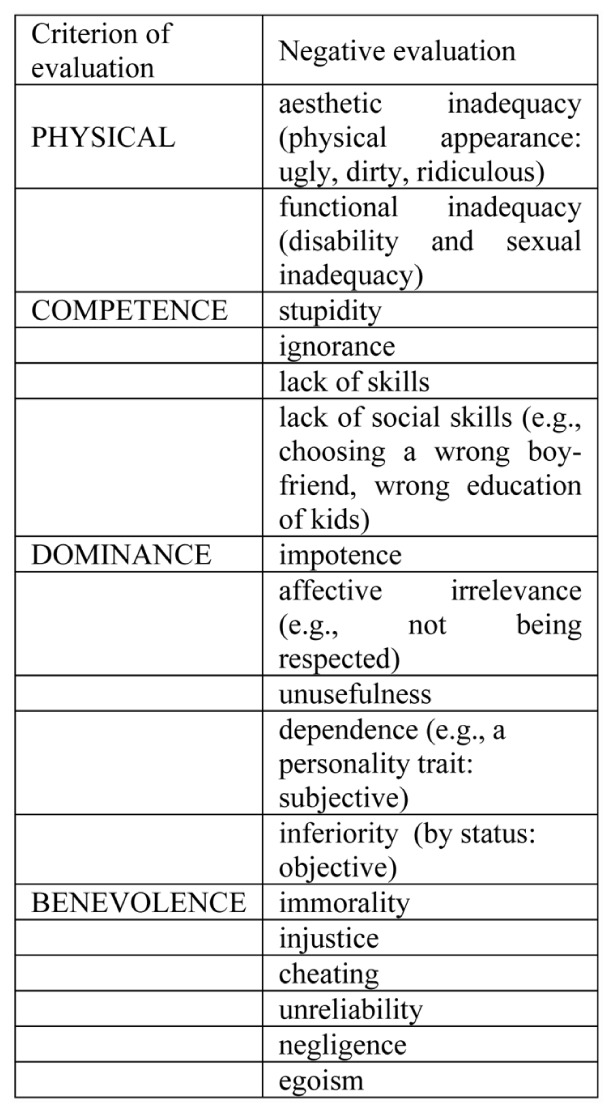
Offensive evaluations.

Is any and every negative evaluation offensive? Probably not: only those evaluations vis-à-vis criteria that we consider important for our image are felt as attacks to it. From this point of view, the discrediting evaluations mentioned by our participants as offensive, in partial analogy with previous works on the discrediting acts in political communication (Poggi et al., 2015) can be classified as in **Figure 2** a physical (aesthetic or functional) inadequacy, plus inadequacy with respect to the criteria of competence (cognitive skill), dominance (power and decisional effectiveness), and benevolence (a moral criterion).

A typical case in which people feel offended is when they are teased or criticized for their PHYSICAL APPEARANCE, based on an aesthetic criterion. But even more offensive it is targeting the functional properties of a person’s physical arrangement: the stygma of handicap makes the person feel humiliated, and when mentioned or alluded to it is offensive, even, surprisingly, when the intention of B is not to offend but, for instance, rather to help (pity is humiliating).

Referred to the criterion of COMPETENCE, participants mention the attribution of stupidity, but also lack of social skills (like being told you are not able to educate your children).

Concerning DOMINANCE, the other’s carelessness is offensive since it tells they consider us irrelevant. We feel totally inconsequential when we are considered or explicitly accused to be useless, but also when people ignore us, or they omit those simple acts that credit us with dignity and deem us as worth respect. Further offensive attacks to our DOMINANCE are when others consider us inferior.

Coming to BENEVOLENCE, we are offended by attributions of immorality, of cheating or unreliability, of negligence or non-compliance with our duty, and finally of selfishness.

#### The Necessary Conditions of Feeling Offended

The real examples of our participants provide the mental ingredients of the offense – its sufficient conditions – as drawn from positive cases. Yet, an euristic way to find out the necessary conditions of a phenomenon is to wonder what happens when that phenomenon fails to occur. So we put two more questions to our participants:

n.19: *Did it happen that someone wanted to offend you but you actually did not feel offended? And if so, why didn’t you?*
and
n.22: *Were there cases when someone felt offended but actually s/he had no reason to feel so? Report one and explain why did s/he felt offended, and why s/he had no reason to feel so.*

From answers to question 19 (someone wanted to offend but you did not feel offended) the following conditions seem necessary for the feeling of offense.

(a) No relevance of the flaw for A (no value sharing)
B’s criterion of evaluation is not considered important by A
18: *per il mio modo differente di agire e pensare.*
(I did not feel offended) due to my different way of acting and thinking.
(b) No truth of the event (no fact sharing)
the event criticized by B does not hold in fact
20: *perchè lei voleva entrare nel nostro gruppo di amiche, e pensava che la sua assenza in quel gruppo fosse causa mia.*
(because she wanted to enter our group of friends, and thought her absence there was caused by me).
(c) No goal of offending in B
B did not really have the goal of wounding A, for instance when simply joking, or when criticism is aimed at advice or benevolent pedagogical goals, or finally when A understands that the reason why B tried to disvalue A was motivated by B’s “self-serving goals”: e.g., out of envy or to establish his superiority over A; therefore, he may “decide” not to get offended as a form of “psychological reactance” (Brehm and Brehm, 1981): the more one wants something from you, the less you do what he wants.
(d) No relevance of B for A
B and B’s judgment are not important for A since it is not important for A to maintain a social-affective relationship with B, or, reciprocally, A does not esteem B.
(e) No touchiness of A
Some participants generally do not attribute a high offensive potential to some kinds of criticism, or they imply that the ease of feeling offended depends on people’s personal sensitivity to others’ judgment, i.e., A’s particular touchiness; others even consider their not being hypersensitive to others’ comments as a matter of moral superiority, often helped by their skills of humor and irony
(f) No dependence of A’s self-esteem from others’ esteem
The clearest protection from feeling offended is being so self-confident as not to be too dependent on other people’s judgment: like implied by this answer:
54: *Ho capito che ciò che importa è come sono e come mi sento io e non come le persone intorno a me mi vedono.*
(I understood that what is important is how I am and how I feel and not how people around see me).

The same conditions drawn from question n.19 pop up from the specular question n.23: if someone felt offended, but in your opinion should have not, why shouldn’t s/he? For some participants, one should not feel offended because the other did not really want to hurt, or was simply joking, or aimed to help (e.g., giving advice, not judging). Moreover, a further necessary condition for really feeling offended is the seriousness of the criticism.

### A Comprehensive Definition of “Feeling Offended”

Since defining an emotion implies finding out the necessary conditions for a person to feel that emotion, we summarize the above analysis in a set of conditions, among which – in a Searle-like manner – we can distinguish (a) preparatory conditions, (b) essential conditions, and (c) aggravating conditions.

Preparatory conditions

A has the goal of a positive image before B
A has the goal of a positive image before third parties C
A has the goal of a positive self-image
A believes that property X is pertinent for his goal of image before B or before third parties C

Essential conditions

B perfoms an Action A
Or else
B omits to perform Action A
A believes that this explicitly communicates or indirectly implies
That B attributes a flaw X to A
X thwarts the image that A wants to project of himself
To B
And/or to third parties C
And/or to him/herself
A believes that X makes him/her inferior to B/C
Or to the category to which A wants others to believe he belongs to.
All of this causes A to feel
a negative image emotion (sadness, displeasure, shame, humiliation)
and/or a negative social emotion toward B (anger or rancor)
a negative emotion of affiliation (inferiority, feeling of exclusion)
a negative emotion of attachment toward B (disappointment about B)

Aggravating conditions

The negative emotion of A is as more dramatic as
The manifestation of A’s flaw is public, i.e.,
A believes that third parties C will come to know about As’ flaw or inferiority
A believes that B’s attack to A’s image is deliberate
A has a low self-esteem
A’s self-image is strongly dependent on the image that others (B and/or C) have of A
For A the goal of having a positive social (possibly affective) relationship to B is important
A esteems B.

### Results: Features, Attributions, Emotions, and Evaluations in Feeling Offended

Data grouped around the above notions were subject to quantitative analysis, by coding them as follows:

(1) *Features of feeling offended*. Each episode was codified, where explicit enough, as to:
  (a) Evaluation criterion (benevolence, dominance, competence, physical).
  (b) Cause (actions, communicative acts, inferred mental states).
  *Actions* were coded as: omission, betrayal, injustice, unfriendly stance, deception, and misunderstanding;
  *Communicative acts* as: increasing level of aggression, silence, refuse, exclusion, negative prediction, detraction, reproaches, negative formal evaluation, criticism, mockery, accusation, slander, aggression, insult.
  *Inferred mental states*, as mistrust or carelessness.
  (c) Personal and relational components: private vs. public, depending on whether the offense was in a personal relationship or before others; and context (family, friends, acquaintances, working relations).
(2) *Causal attribution*. whether the cause of the feeling was oneself, another person, or simply something happened (rated on a *5-points* Likert scale: *1 = nothing at all, 4 = very much*); stability of the attribution was coded, where possible, from the personal recall of episodes; in case of transitory features the attribution was “unstable,” unlike cases where something cannot be changed, coded as “stable.”
(3) Emotions associated to feeling offended (on a *5-points* Likert scale: *1 = nothing at all, 4 = very much*).
(4) Evaluation of the “offender” and possible forgiveness after the offense (on a *5-points* Likert scale: *1 = nothing at all, 4 = very much*).

#### Features of Feeling Offended

(a) Types of evaluation

Feeling offended is an emotional state caused by a communicated (direct) or inferred (indirect) negative evaluation, conveyed by (1) an action, (2) a communicative act, or (3) the other’s inferred mental state. The evaluations that most likely cause the offence (see **Table 1**) concern *dominance* (37.6%) and *competence* (35.8%), and to a lesser extent *benevolence* (14.7%) and *physical appearance* (11.9%) (χ^2^ = 2.89; *p* < 0.05). The specific evaluations of lack of dominance include dependence, helplessness, inferiority, uselessness, and especially irrelevance, while lack of competence ranges from negligence to stupidity, to social competence (to be a good mother). In our database cases referred to female sexuality or masculinity are quite rare (unlike in Cohen et al., 1996; Mosquera et al., 2002).

**Table 1:** Evaluation types ^∗^ emotions associated to feeling offended.

	Pride	Shame
Benevolence	2,19 (0.3)	1,75 (0.7)
Competence	2,95 (0.4)	2,42 (0.6)
Dominance	2,71 (0.8)	1,55 (0.9)
Physical features	1,92 (0.4)	3,23 (0.6)

##### Type of evaluation ^∗^ emotions

Even if the most frequent type of evaluation conveyed by offenses concerns the dominance criterion, this is not the most emotionally arousing: crossing type of evaluation with emotions, the negative evaluations of physical appearance result to cause *shame* significantly more than those on dominance, benevolence, and competence, respectively [3.23 vs. 1.55, 1.75, and 2.52; *T*-test (105); *p* < 0.005]. Differently, when conveying lack of competence and dominance offense causes a *pride* reaction more likely than when on benevolence and physical appearance [2.95 and 2.71 vs. 2.19 and 1.92; *T*-test (105); *p* < 0.05].

(b) Causes of the offense: actions, communicative acts, and mental states

The negative evaluations that cause a state of offense are conveyed, in our participants’ autobiographic reports, in three ways: actions or behaviors, explicit communicative acts, and mental states inferred by the offender. The most frequent (see **Table 2**) are *communicative acts* (60%), then *behaviors* (24%), and third the *other’s implied mental states* (16%). If thus 60% of the offenses are borne through communicative acts, the remaining 40% are simple behaviors and inferred mental states, coded as indirect evaluation (indirectness: 45% in private vs. 27% in public contexts; χ^2^ = 3.67; *p* < 0.05). People not only admit they can feel offended by non-communicative actions or even non-actions (as in case of an omission or an unfriendly stance of the offender), but also that in communicative acts too the negative evaluation can be communicated indirectly.

**Table 2:** Causes ^∗^ emotions associated to feeling offended.

	Shame	Bitterness
Communicative acts	2,38 (1.0)	3,43 (0.2)
Behaviors	1,70 (0.8)	3,95 (0.5)
Inferred mental states	1,45 (0.9)	3,90 (0.6)

The most quoted communicative acts causing offense are simple criticism (24%), insult (16%), accusation (15%), and reproach (8%), mostly directed toward the target (but participants’ descriptions remark that also communicative acts in absence of the target can be a possible cause); other offensive communicative actions are mockery (16%), slander (5%), and calumny (2%). Further possibilities are silence, refuse, or exclusion.

Among offensive behaviors participants first include omissions (27%) and betrayals (27%), then injustice (17%), unfriendly stances (13%), deception or taking advantage (10%), and misunderstanding (6%).

Finally, the underlying mental states attributed to B that offend A are mistrust and carelessness (48% vs. 52%).

##### Causes ^∗^ emotions

As to relations between offensive events and emotions, communicative acts cause *shame* more then do behaviors or inferred mental states (2.38 vs. 1.70 and 1.45, respectively) possibly because they can be heard by other persons, whereas *bitterness* is significantly caused more by behaviors and inferred mental states than by overt communicative acts [respectively, 3.95 vs. 3.90 and 3.43; *T*-test (112); *p* < 0.05]. A hypothesis to account for this is that the stronger cognitive work required for inferring indirect evaluations more easily induces the victim to the continuous rumination typical of bitterness (Poggi and D’Errico, 2010).

(c) Components of feeling offended: a private/personal/relational vs. public injury.

Feeling offended has been generally studied in relation to negative communicative acts (McCullough, 2000; Mosquera et al., 2002), but when participants are free to remember autobiographical episodes a wider range of causes emerge, such as omissive behaviors, unfriendly stance or even silence, that can be offensive when they lead participants to feel a gap between personal expectations and real acts (Zander, 1976). When coding narrative episodes in our survey (108 cases/129), we found that, taken together, behaviors, communicative acts, and implied mental states are mainly related to *personal and private relations* (64%), where the presence of other persons looks almost irrelevant; therefore in our study feeling offended is associated to public or honor code (36%) to a much lesser extent than in previous studies (Mosquera et al., 2002). People can feel deeply offended when they understand that someone, directly by words or indirectly by actions or omissions, reveals/shows an unexpected negative evaluation of them, whether or not before others.

#### Causal Attributions

Participants attribute their feeling offended to others, oneself, or simply the situation (“something happened”). Results highlight that this emotional state is strongly associated with a strong attribution of responsibility to the other person (3.58; *p* < 0.05) or, to a lesser extent, to “something happened” (3.38), with personal attribution much less frequent (1.95). Furthermore, participants attribute their feeling offended more to the other person’s intention (3.31; *p* < 0.05) than to contextual factors (2.98).

##### Causal attribution ^∗^ emotions

The causal attribution of a received offense can affect different types of emotions of both image and self-image (shame and guilt) and even those linked to the goal of survival (fear); if generally when feeling offended a person tends to attribute responsibility to the offender, when an internal attribution is acknowledged, emotions of fear, shame, and guilt significantly increase (**Table 3**). Fear is an interesting case: it may be associated with a more stable trait that the offended person acknowledges to herself and that she probably recognizes as a cause of future negative experiences. For example, if she acknowledges that her body as a stable source is repeatedly subject to negative evaluation (see below, type of evaluation ^∗^ emotion) she may anticipate the fear that this evaluative process will accompany her for the course of her life, thus becoming an emotion associated not only with the image of herself (as in the case of shame or guilt), but even to survival goals. Likewise, shame and guilt are related to an internal attribution of the offense. Furthermore, the external conditions taken as responsible correlate with fear, possibly because they become less controllable by participants, hence potentially threatening (**Table 3**).

**Table 3:** Correlations causal attribution ^∗^ emotions associated to feeling offended.

		Something I	Something the	Something
		have done	other person	that
			has done	happens
Fear	Pearson Corr.	**0,311^∗∗^**	0,006	**0,182^∗^**
	Sign.	0,000	0,951	0,040
	*N*	127	127	127
Shame	Pearson Corr.	**0,266^∗∗^**	–0,051	0,049
	Sign.	0,002	0,573	0,588
	*N*	127	127	127
Guilty	Pearson Corr.	**0,175^∗^**	–0,001	0,046
	Sign.	0,048	0,992	0,604
	*N*	127	127	127

*^∗∗^Correlation sign at 0,01 (two-way codes). ^∗^Correlation sign at 0,05 (two-way codes). Significant values in bold.*

Getting more into the dimensions of causal attribution, feeling offended can be associated mostly to an unstable cause (73% vs. 27%), i.e., something that can be changed in time; unstable causes mainly relate to competence or dominance (37.5% and 41.7%) unlike stable causes, significantly (χ^2^ = 19.00; *p* < 0.00) referred to physical evaluation (37%). The stable causes in feeling offended are recalled more by women than men [(76% vs. 24%); χ^2^ = 3.65; *p* < 0.05]. Stable attributions have an important effect on shame and pride: a *t*-test analysis points out that with negative evaluations attributed to stable causes the offended person feels significantly more shame and less pride than with unstable ones (2.83 vs. 1.85; *t* = 3.67; *p* < 0.00; 2.07 vs. 2.78; *t* = 2.64; *p* < 0.05). This can be considered a very strong factor in explaining shame as tested also by a significant regression with gender, self-esteem, and stability of attribution where this last one is the best predictor, thus demonstrating that when the cause of the negative evaluation cannot be changed, one feels more intensely offended (β = 0.27; *p* < 0.00).

The context of the offense, private or public, and the relationship with the offender can differentiate the internal processes of causal attribution in that, while with friends and acquaintances people tend to attribute the responsibility of the offense mostly to the other (*r* = 425; *r* = 0.306; *p* < 0.00) or to something happened (*r* = 0.283; *r* = 0.393; *p* < 0.00), with co-workers only an external attribution is present (*r* = 273; *p* = 0.002), while in family relations people tend to shift responsibility to oneself (*r* = 0.237; *p* < 0.007). So only when the relationship is closer people are likely to make a personal exam of the all possible responsibilities, otherwise the tendency is to attribute one’s own state to an external cause (**Table 4**).

**Table 4:** Correlations types of relationship ^∗^ causal attribution.

		Familiar	Friends	Acquaintances	Working relations
Something I have done	Pearson Corr.	**0,237^∗∗^**	0,088	0,077	0,076
	Sign.	0,007	0,318	0,388	0,401
Something the other person has done	Pearson Corr.	**0,298^∗∗^**	**0,425^∗∗^**	**0,306^∗∗^**	**0,273^∗∗^**
	Sign.	0,001	0,000	0,000	0,002
Something it happens	Pearson Corr.	**0,423^∗∗^**	**0,283^∗∗^**	**0,393^∗∗^**	0,160
	Sign.	0,000	0,001	0,000	0,076

*^∗∗^Correlation sign at 0,01 (two-way codes). ^∗^Correlation sign at 0,05 (two-way codes). Significant values in bold.*

The relation between causal attribution of the offense and the types of personal relationships can in turn affect the associated emotions of this emotional state; the closer the relation the more inner focused the emotions: in-family offense in fact is mainly associated to sadness (*r* = 0.242; *p* < 0.007) and sense of guilt (*r* = 0.171; *p* < 0.05); with friends, to fear and sense of guilt; with acquaintances shame can prevail (*r* = 0.248; *p* < 0.05); just in professional relations feeling offended is associated with anger (*r* = 0.192; *p* < 0.032) and bitterness (*r* = 0.187; *p* < 0.038), both intrinsically connoted by a sense of injustice (Izard, 1975; Poggi and D’Errico, 2010; D’Errico and Poggi, 2014) (**Table 5**).

**Table 5:** Correlations types of relations ^∗^ emotions associated to feeling offended.

		Anger	Sadness	Fear	Shame	Bitterness	Sense of guilt
Family	Pearson Corr.	0,076	**0,242^∗∗^**	**0,231^∗∗^**	–0,015	0,082	**0,171^∗^**
	Sign.	0,395	0,007	0,009	0,866	0,357	0,050
Friends	Pearson Corr.	0,105	0,143	**0,240**^∗∗^	0,114	0,006	**0,204^∗^**
	Sign.	0,237	0,108	0,006	0,197	0,948	0,021
Acquaintancs	Pearson Corr.	0,051	0,104	0,092	**0,248^∗∗^**	0,082	0,151
	Sign.	0,572	0,250	0,303	0,005	0,360	0,092
Working relations	Pearson Corr.	**0,192^∗^**	0,087	0,078	0,091	**0,187^∗^**	0,015
	Sign.	0,032	0,343	0,392	0,314	0,038	0,867

*^∗∗^Correlation sign at 0,01 (two-way codes). ^∗^Correlation sign at 0,05 (two-way codes). Significant values in bold.*

### Results: Personal and Relational Factors

*Gender differences.* When gender analysis is included, interesting differences emerge. The recalled contexts of feeling offended are mainly family (3.08), friends (3.03), and to a lesser extent acquaintances (2.30); but the recalling focused on family and friends is more frequent in women than in men [*t*-test (129); *p* < 0.05], coherently with the expectations of care generally assigned to females (Burr, 2002): women, who are expected to invest more affectively, are also more likely to feel offended. Moreover, with acquaintances and co-workers, gender differences are not significant. We can therefore state that offenses leave traces mostly within closer relations (**Table 6**).

**Table 6:** Type of relation ^∗^ gender.

	Woman		Men	
	*M*	*SD*	*M*	*SD*
Family^∗^	3.23	0.99	2.84	1.1
Friends^∗^	3.20	1.0	2.76	1.3
Acquaintances	2.39	1.0	2.16	1.0
Work	2.58	1.1	2.47	1.2

**N* = 129; *p* < 0.05.*

When relating gender to reported emotions, ones of opposite arousal emerge: while women tend to express mainly sadness (*p* < 0.05) and bitterness when feeling offended, in men anger and pride prevail (*p* < 0.05); when men feel offended they react by activating themselves as if being mistreated – directly or not – finally assuming masculine and dominant roles. For males, then, feeling offended looks more associated with a violation of honor rules (Mosquera et al., 2002) and with image before others than for females (**Table 7**).

**Table 7:** Emotions associated to feeling offended.

	Woman		Men		Total
	*M*	*SD*	*M*	*SD*	
Anger^∗^	3.99	1.0	4.24	1.1	4.09
Sadness^∗∗^	3.82	1.1	3.41	1.2	3.66
Fear	1.56	0.9	1.53	0.8	1.55
Pride^∗^	2.52	1.3	2.88	1.6	2.66
Shame	2.11	1.2	1.88	1.2	2.02
Bitterness^∗^	3.87	1.3	3.47	1.3	3.67

**N* = 129; ^∗^*p* < 0.05; ^∗∗^*p* < 0.001.*

### Effects of Feeling Offended on the Self and the Relationship with the Other

*Effects of feeling offended*. From our ANOVA analysis with repeated measures it emerged that the positive image of the offending person, as expected, worsens after the offense [decreasing from 3.63 to 2.26; *F*(1,123) = 148.00; *p* < 0.000]. However, the positive assessment of the other person is a factor in protecting the relationship, since it is positively correlated to, and increases the possibility of forgiving the offense (*positive evaluation before offense ^∗^ forgiveness*: *r* = 0.332; *p* < 0.000; *positive evaluation after the offense ^∗^ forgiveness*: *r* = 0.465; *p* < 0.000).

The possibility of forgiving is, however, differently associated with the emotions felt in the offense: emotional experiences that correlate negatively with positive evaluation after the offense (*r* = -0.360; *p* < 0.000) and with the possibility of forgiving include anger (*r* = -0.352; *p* < 0.000), but clearly also “memory” emotions such as the rumination of bitterness (*r* = 0.230; *r* = -0.179; *p* < 0.04) and rancor (*r* = 0.287; *r* = -0.362; *p* < 0.000); both anger and bitterness are more frequent in working contexts (**Table 5**), and presumably the positive evaluation of the other and possibility to forgive after the offense are less likely when the offense is made within working relations.

The correlation *emotions* ^∗^ *effects of offense* might also account for the fact that women, who reported more sadness and sense of guilt, tend to forgive significantly more than men (70% vs. 30%, χ^2^ = 8.818; *p* < 0.012), in line with other studies (Miller et al., 2008). The correlations with self-conscious emotions such as pride and shame are less relevant for the effects of feeling offended, where rather basic dimensions prevail linked to violated rights or personal expectations. An interesting result is the positive correlation between guilt and positive evaluation of the offender: when one attributes responsibility to oneself, the image of the other is preserved (**Table 8**).

**Table 8:** Offender’s positive image and forgiveness ^∗^ emotions associated to feeling offended.

		Positive image	Positive image	Forgiveness
		(before offense)	(after offense)	
Anger	*R*	–0,087	–**0,360^∗∗^**	–**0,352^∗∗^**
	Sign.	0,327	0,000	0,000
Sadness	*R*	**0,202^∗^**	–0,008	0,034
	Sign.	0,023	0,927	0,699
Fear	*R*	–0,080	–0,098	**-0,179^∗^**
	Sign.	0,372	0,270	0,042
Pride	*R*	–0,009	–0,066	–0,119
	Sign.	0,919	0,460	0,176
Shame	*R*	–**0,225^∗^**	–0,064	–0,013
	Sign.	0,011	0,471	0,884
Bitterness	*R*	–0,091	–**0,230**^∗∗^	–**0,179^∗^**
	Sign	0,308	0,009	0,042
Rancor	*R*	–0,105	–**0,287^∗∗^**	–**0,362^∗∗^**
	Sign.	237	0,001	0,000
Guilty	*R*	0,142	**0,224^∗^**	0,080
	Sign.	0,111	0,011	0,364

*Perdono no = l si = 2 punteggi piu alti aumenta il perdono. ^∗∗^Correlation sign at 0,01 (two-way codes). ^∗^Correlation sign at 0,05 (two-way codes). Significant values in bold.*

The positivity of the other’s image worsens significantly in relation to the type of relation with the person who offended; basically, in a personal relation the other’s image is comprehensibly more positive, but after the offense positivity strongly decreases, much more than in public contexts. [Interaction effect *time^∗^ type of relation F*(1,103) = 7,145; *p* < 0.01)] (**Table 9**).

**Table 9:** Positivity of other image ^∗^ relation type.

	PERS		PUB	
	*M*	*SD*	*M*	*SD*
Pre	4.22	0.97	2.73	1.2
Post	2.53	1.2	1.81	0.9

**N* = 129.*

### Self-Esteem and Feeling Offended

The second aim of our work was to investigate the role played by personal traits, in particular self-esteem, on the feeling of offense, conceptualized in a comprehensive way, including explicit and implicit causes. While we have seen that offense results in lowering the self-esteem of the offended person, here self-esteem is not considered as a possible outcome but as an antecedent of “feeling offended,” hence as a possible protection factor to support people from aggressive communicative contexts. In this perspective self-esteem is viewed as a buffer (Andt and Goldenberg, 2002), a coping potential against negative emotional reactions to feeling offended. Furthemore, we will explore the potential relation between self-esteem and associated emotions, by also assessing gender differences in feeling offended. Such analysis can be linked to well-known studies stating how women have a lower self-esteem than men (Baumeister et al., 2003), so we could expect persons with low-self-esteem to live offenses with more negative low arousal emotions (sadness and bitterness), internal attributions, and rumination (sense of guilt and rancor) than people with high self-esteem.

#### Measures

##### Self-esteem

The Rosenberg Self-esteem Scale (Rosenberg, 1965) consists of 10 statements (1 = strongly disagree, 7 = strongly agree). Half of the items are negatively formulated and were therefore coded in reversed form. Example items are “I feel that I am a person of worth at least on an equal basis with others” and “I certainly feel useless at times.”

A factorial analysis on the self-esteem scale confirmed a single-factor scale structure whose items saturate greater than 0.40 on a single factor and good internal reliability (Cronbach’s α = 0.82). The self-esteem score (min = 1.8, max = 4, Me = 3.17, DS = 0.48) was calculated. For that index, subsequently, the median value was used as discrimination to distinguish high and low levels of self-esteem.

##### Dependent measures

(1) *Causal attribution* – oneself, other, something happened – on a five-points Likert scale, *1 = never, 5 = very often*).
(2) Emotions associated to feeling offended (five-points Likert scale; *1 = nothing at all, 5 = very much*).
(3) Evaluation of the “offender” and forgiveness after offense (five-points Likert scale; *1 = nothing at all, 5 = very much*).

#### Results

Crossing gender and self-esteem, our data confirm that men in the average have a higher self-esteem than women [(67% vs. 33%); χ^2^ = 4,622; *p* < 0.030].

##### Causal attribution

Both internal attribution (2,32 vs. 1,64; ANOVA: *F*(1,131) = 4,147; *p* < 0.000) and external attribution to an event (something happened) (3,66 vs. 3,15; *F*(1,131) = 2,813; *p* < 0.006) are higher in case of low self-esteem; when people give a negative global evaluation of the self (Crocker et al., 1993), even when receiving offense they more likely attribute it to themselves or have a more fatalistic attribution, somehow considering the possibility they contributed to that failure/offense. No significant difference in the attribution to the other (**Table 10**).

**Table 10:** Causal attribution ^∗^ self-esteem.

	Low		High	
	Mean	*SD*	Mean	*SD*
Something I have done^∗^	2.32	1.1	1.64	0.77
Something the other person has done	3.68	0.93	3.50	1.0
Something it happens^∗^	3.66	1.04	3.15	1.01

*^∗^*p* < 0.05.*

##### Emotions associated to feeling offended

Participants with high self-esteem express lower levels of negative low arousal emotions when feeling offended: lower sadness, shame, bitterness, rancor, and sense of guilt (ANOVA significant *p* < 0.05; **Table 11**); differently, people with low self-esteem feel higher levels of negative low arousal emotions, but also fear, showing that their negative global evaluation leads them to experience lack of internal resources to face the received offense (**Table 11**).

**Table 11:** Associated emotions of feeling offended ^∗^ self-esteem.

	Low SE		High SE	
	*M*	*SD*	*M*	*SD*
Sadness^∗^	3.81	1.1	3.37	1.3
Fear^∗∗^	1.80	1.0	1.32	0.07
Shame^∗∗^	2.27	1.2	1.73	1.1
Bitterness^∗∗^	3.78	1.1	3.42	1.3
Rancor^∗∗^	2.68	1.4	2.11	1.3
Sense of guilt^∗∗^	1.78	1.1	1.38	0.07

**N* = 129; ^∗^*p* < 0.05;^∗∗^*p* < 0.01.*

##### Mediational analysis between gender, self-esteem, and sadness/shame in feeling offended

To outline the relation between gender, self-esteem, and negative emotions like shame and sadness in feeling offended, we performed six mediational analyses (three for sadness and three for shame). To test the mediational hypothesis, a series of regression analyses was performed following the procedures outlined by Baron and Kenny (1986).

This procedure consists of a three-step series of regression analyses. In the first regression, the independent variable is associated with the dependent variable; in the second regression, the independent variable has to be associated with the hypothesized mediator. The third phase consists of a regression where the effects of both independent variable and mediator on the dependent variable are tested. Mediation is demonstrated when the addition of the mediator variable into the third regression equation substantially decreases or eliminates the previously significant relation between the independent variable and the dependent variable. The relation involved in the mediations was also tested by means of Sobel test (*T*: 1,69; *p* < 0.05 for sadness and *T*: 2,33; *p* < 0.05 for shame).

**FIGURE 3:**
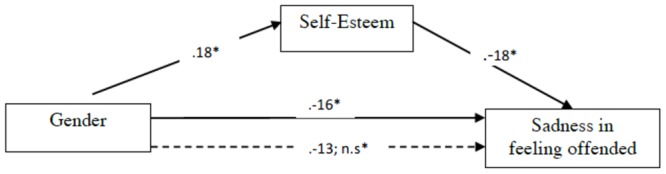
Mediational regression analyses of gender and self-esteem on sadness associated to feeling offended. Gender variable was processed as a progressive number 1 = woman, 2 = man, so the positive relation must be read as follows: when gender increases, sadness in feeling offended decreases ^∗^*p* < 0.05. Solid lines between variables denote direct paths between two variables. Dotted lines denote paths when self-esteem is included as mediator. Values denote standardized beta weights.

**FIGURE 4:**
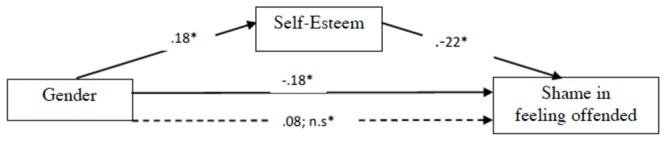
Mediational regression analyses of gender and self-esteem on shame associated to feeling offended. Gender variable was processed as a progressive number 1 = woman, 2 = man, so the positive relation must be read as follows: when gender increases, shame in feeling offended decreases ^∗^*p* < 0.05. Solid lines between variables denote direct paths between two variables. Dotted lines denote paths when self-esteem is included as mediator. Values denote standardized beta weights.

**Figures 3, 4** show how the experimental condition (1: women, 2: men) is significantly related to self-esteem, with men showing higher self-esteem (β: 0.018). Self-esteem correlates negatively with sadness and shame in feeling offended (β: -0.018; β: -0.022): the more self-esteem, the less sadness and shame in feeling offended (**Figures 3, 4**), and their mediation also lowers the direct relation between gender and sadness and between gender and shame (*p*: n.s.), being a good moderator. So, women are more likely to have a lower self-esteem than men and the relation between gender and self-esteem makes them more likely to feel sadness and shame when feeling offended.

This mediation data highlight the importance of detecting personal features in the feeling of offense, especially when it comes to stable traits like gender and self-esteem, since low self-esteem blatantly contributes to a significant negative reaction in terms of sadness and even shame, by potentially reinforcing a negative evaluation of oneself. Reading this result on the other side, we conclude that self-esteem can be considered a good “buffer,” across different relations, in presence of offensive words, actions, and omissions.

### Discussion

Previous literature on the offense has viewed it almost exclusively as an attack to the public image of a person, to her own, or her family’s honor. But the autobiographic episodes told by our participants witness that the attack to the person’s self-image is much more frequent, offensive, and serious than public loss of face, since it can deeply hurt and jeopardizes her relationship with the offender.

Moreover, such a blow to a person’s image and self-image is hit not only by explicit statements of a person’s flaws or mistakes (60% of offensive actions), including criticisms, accusations, reproaches, and insults, (only 16%, unlike in Mosquera et al., 2002), but also by a 40% of so-called indirect offenses, i.e., non-communicative behaviors like betrayal (27%) or omission (27%), which may be interpreted by the target as cue to the other’s carelessness or distrust, in turn pointing at a deeply negative evaluation of one’s self. Unexpectedly, the offense most typically takes place in a personal context (64%), not necessarily in presence of other people.

Feeling offended is then mainly an intimate condition in which the offended person, especially in her family relationships, may attribute a part of responsibility to oneself (see the correlation between “something I have done” and family context, β = 0.237; *p* < 0.05), whereas with aquaintances and working relations one tends to attribute the cause of the offense to the other.

Like in Mosquera et al. (2002) study, a gender difference holds in that women, probably due to their higher investment in affective relations (Burr, 2002), tend to feel offended more in family or friend contexts. Further, while men’s affective response is more frequently of pride and anger, women live the offense in more ego-focused ways, feeling more sadness, shame, and bitterness – an emotion implying longlasting rumination. Since the offense is more frequent in family contexts, if women attribute at least part of responsibility to themselves, making themselves guilty, this may make the offense itself more bearable, since the previously positive image of the offender does not change so much (beta-correlation = 0.224; *p* < 0.05). Two emotions instead which do not favor forgiveness and worsen the other’s image are anger (β: -352; β: -360) and rancor (β:-287; β: 362). When feeling offended is caused by a sense of injustice, forgiveness may become more difficult, mostly in non-intimate relations where the other’s positive image is generally lower than in intimate ones; but in intimate relations feeling offended can strongly worsen the image of the other (**Table 9**) and cause deep sadness reactions (**Table 8**).

An offense is an injury not to our body but to our soul; as claimed by a participant in our survey,“a moral injury that always leaves a scar, more or less visible, more or less deep, but anyway a scar.” In our terms, offense is a negative evaluation explicitly communicated or implied by a communicative act, an omission, or other behavior directed to or concerning a target. When referred to a stable trait it can elicit a shameful reaction, being closer to a self-evaluation and possibly more pervasive of the Target’s image (as tested by regression with stability attribution as best predictor). This feature distinguishes insult from other acts of discredit: while a criticism or a reproach may concern a single *action* that the Target has done in one case but might amend in future, being unstable and controllable, less inhesorable, and permanent, an insult claims a negative *property* of the Target so stable and out of control as to become definitorial of the Target (Poggi et al., 2015). Telling you “you did it wrong” may be unpleasant, but not offensive; telling “You are stupid” or “You are A liar” is an insult, hence definitely an offense. This is why insult is a prototypical cause of offense: criticism, accusation, silence, omission, carelessness are more seriously offensive to the extent to which the negative evaluation explicitly understood or simply inferred is read as permanent and unamendable. Criticism may not simply mean “you did this wrong now” but be interpreted as “you ARE incompetent,” and we found that this interpretation is as more likely as the Target’s self-esteem is lower and more conducive to self-confirmation. Since inferences are drawn from previous beliefs, the more I am convinced of my low value, the more easily can I infer a pervasive negative evaluation from even an innocent remark.

As to the relation between feeling offended and self-esteem our data suggest a difference between two types of emotional reaction to the feeling of offense, that we might call a “proud” and a “hangdog” attitude: the former with prevailing emotions of anger and indignation, the latter charaterized by shame, self-blame, sadness, depression. The former tends to occur with unjust criticism or accusation: the Target does share the Offender’s criterion of evaluation, but does not agree that s/he did that wrong action or was the wrong way (s/he does not share factual knowledge), so the reaction is a snapshot of pride before the Offender, as if communicating: I am angry and indignant for this unjust accusation, which reveals distrust in me, ruling out the possibility of simply misunderstanding my behavior; it is not worthwhile to depend on such a person who does not acknowledge my worth. In this case the Target prefers to refrain from her possible previous dependence on the Offender, and to give up any relationship with him. The lack of fact sharing is favored by the Target’s confidence in herself, her values, and her reality perception, that make part of her self-esteem.

In the other case the Target, besides convincingly sharing the Offender’s criterion of evaluation, ends up sharing factual knowledge too, so she can but acknowledge her wrong. In fact, having a low self-esteem implies a constellation of consequences: on the cognitive side, low confidence in one’s own assumptions (uncertain beliefs about whether she or the Offender were right in their perception); on the affective side, higher dependence on the Other and the relationship with him: hence, more displeasure in breaking the relationship, more sadness both for the mourn of losing the other, and for one’s own further nicked self-esteem.

Feeling offended is an adaptively relevant emotion because it monitors the goals of image and self-image, in the same vein as shames does. While you are ashamed when you simply believe or fear others to make up a negative evaluation of you, you feel offended when such evaluation becomes in some way actual and overt, not only when it is publicly claimed in front of others, but also when you think it can be inferred from the other’s behavior or non-behavior.

All negative emotions have the function to alert the subject that an important goal is at risk of being thwarted, and to induce immediate reactions aimed at a repair.

Here, the very pain of feeling offended alerts us that our goals of image and self-image are challenged by the other, but while in a proud reaction the result may have a function to claim our own personal value (though sometimes at the cost of breaking the relationship), in a person with low self-esteem the wound could remain open by leading to a negative processing finally confirming such low value, hence inducing a more passive emotional reaction.

## Conclusion

This work has presented a survey study investigating nature, contexts of occurrence, affective, and relational effects of the feeling of offense: an emotion that monitors our goals of image and self-image, that are so basic for the human animal as to make the connected emotions really basic too. Based on a content analysis of participants’ answers, within a socio-cognitive model of emotions that argues for an adaptive function of each of them, the study has singled out the mental ingredients of feeling offended, its necessary and aggravating conditions, the causes of offense, and resulting offensive evaluations.

An offense is a blow to our image and our self-image, which makes us feel offended. Feeling offended is a negative emotion caused by a communicative or non-communicative act or omission of another person that explicitly points at or implies a negative property of the Target, who generally shares the other’s criterion of evaluation, cares his/her judgment, and wants or used to entertain a positive relationship with him/her. This triggers emotions of anger, disappointment, bitterness, rancor toward the other, it often causes the break of the relationship, and lowers the Target’s self-esteem. Unlike previous works mainly viewing offense as a blow to a person’s honor and public image, our study has shown that people feel offended more in personal than in public relationships, and that its causes are not only insult or criticism, but also the other’s arrogance or betrayal, carelessness, or distrust, since they imply offensive evaluations ranging from ignorance to lack of skill and stupidity, to immorality or cheating, inferiority, irrelevance, ugliness, or handicap. Responsibility for the offense is attributed not only to the other, but sometimes to oneself, mainly by women, who pay more attention to personal relationships, and by persons with low self-esteem. People may show two reactions to feeling offended: a proud reaction, where one rejects the negative evaluation with anger and indignation, considering the criticism unjust, possibly breaking the relationship; and a “hangdog” reaction, where one takes part of the responsibility for the offense, and feels sadness, guilt, even fear, further lowering one’s self-esteem. Yet, a previous high self-esteem, seen as an antecedent of the offense, works as a protection factor to support people from aggressive communicative contexts, a coping potential against negative emotional reactions to feeling offended.

This study can be framed within research on the emotions of image and self-image. The complex field of the relations among self-esteem, gender, and the feelings of offense is here explored only on the personal ground, neglecting its important historical–cultural aspects. Future research might investigate it through cross-cultural comparison. Moreover, the specific emotion of feeling offended might be compared with close but subtly different emotional states like feeling humiliated, demoralized, mortified, and taking it personally. In this connection, two aspects of feeling offended might be distinguished, one more linked to challenge to one’s power and image, and one to one’s attachment relationships.

## Author Note

The paper is due to both authors to the same extent, though the order of names mainly recalls responsibility for the very first idea of the research topic.

## Ethics Statement

The methodology of the study has been approved by the Ethical Committee of Roma Tre University, in accordance to the Ethical Principles of research involving Human participants.

## Author Contributions

The two authors are responsible for the ideation of the whole article, discussion, and conclusion. FD is in particular responsible for related studies review, quantitative data analysis, and interpretation of results. IP is responsible for topic ideation, introduction and theoretical part, and qualitative data analysis. They both ensure the integrity and accuracy of the work.

## Conflict of Interest Statement

The authors declare that the research was conducted in the absence of any commercial or financial relationships that could be construed as a potential conflict of interest. The handling Editor declared a shared affiliation, though no other collaboration, with the authors.

## Footnotes

1 In English *injury* means both “wound” and “insult,” and in Italian, the adjective *offeso/offesa*, beside referring to one who feels insulted, outraged, also refers to a wound or disability (e.g., “muoveva faticosamente il braccio offeso” = he labored hard on the offended arm). The English and Italian root *offend-* derives from Latin *offendere*, “to strike”.
